# *In Vitro* and *in Vivo* Antitumoral Effects of Combinations of Polyphenols, or Polyphenols and Anticancer Drugs: Perspectives on Cancer Treatment

**DOI:** 10.3390/ijms16059236

**Published:** 2015-04-24

**Authors:** Massimo Fantini, Monica Benvenuto, Laura Masuelli, Giovanni Vanni Frajese, Ilaria Tresoldi, Andrea Modesti, Roberto Bei

**Affiliations:** 1 Department of Clinical Sciences and Translational Medicine, University of Rome “Tor Vergata”, Rome 00133, Italy; E-Mails: kurk84@gmail.com (M.F.); monicab4@hotmail.it (M.B.); ilaria3soldi@hotmail.com (I.T.); modesti@med.uniroma2.it (A.M.); 2 Department of Experimental Medicine, University of Rome “Sapienza”, Rome 00164, Italy; E-Mail: Laura.masuelli@uniroma1.it; 3 Dipartimento di Scienze Motorie, Umane e della Salute, Università di Roma, Foro Italico, Rome 00194, Italy; E-Mail: frajese@hotmail.com

**Keywords:** polyphenols, bioavailability, carcinogenesis, anticancer drugs, nanotechnology

## Abstract

Carcinogenesis is a multistep process triggered by genetic alterations that activate different signal transduction pathways and cause the progressive transformation of a normal cell into a cancer cell. Polyphenols, compounds ubiquitously expressed in plants, have anti-inflammatory, antimicrobial, antiviral, anticancer, and immunomodulatory properties, all of which are beneficial to human health. Due to their ability to modulate the activity of multiple targets involved in carcinogenesis through direct interaction or modulation of gene expression, polyphenols can be employed to inhibit the growth of cancer cells. However, the main problem related to the use of polyphenols as anticancer agents is their poor bioavailability, which might hinder the *in vivo* effects of the single compound. In fact, polyphenols have a poor absorption and biodistribution, but also a fast metabolism and excretion in the human body. The poor bioavailability of a polyphenol will affect the effective dose delivered to cancer cells. One way to counteract this drawback could be combination treatment with different polyphenols or with polyphenols and other anti-cancer drugs, which can lead to more effective antitumor effects than treatment using only one of the compounds. This report reviews current knowledge on the anticancer effects of combinations of polyphenols or polyphenols and anticancer drugs, with a focus on their ability to modulate multiple signaling transduction pathways involved in cancer.

## 1. Introduction

Polyphenols, a large group of compounds ubiquitously expressed in plants, are secondary metabolites that play various roles in host defense against pathogens, ultraviolet radiation, and signal transduction [1]. Polyphenols are present in foods and beverages of plant origin (fruits, vegetables, cereals, herbs, spices, legumes, nuts, olives, chocolate, tea, coffee, and wine) and are the most abundant antioxidants in the human diet [2]. Epidemiological studies have shown that a diet rich in polyphenols can prevent a wide variety of human diseases. Polyphenols show many beneficial effects on human health including antimicrobial, anti-inflammatory, antiviral, anticancer, and immunomodulatory activities [3,4,5,6,7,8].

Carcinogenesis is a multistep process that causes the progressive transformation of a normal cell into a cancer cell [9]. Malignant transformation is due to the overexpression or hyperactivation of genes that promote cell survival and proliferation (oncogenes) or to the loss of expression or functional inactivation of genes that control cell growth (tumor suppressor genes). As a result, while normal cells are responsive to exogenous stimuli that control their growth and survival, cancer cells can grow in the absence of exogenous signals and become unresponsive to negative regulators of growth and survival. In addition, overexpression of growth factors and/or their receptors leads to constant activation of downstream signaling pathways, promoting the growth and survival of cancer cells [9,10,11,12].

Signal transduction pathways involved in carcinogenesis often interact with each other, enhancing oncogenic signals that trigger the malignant phenotype of cells [3,10]. For example, cross-talk between the signaling pathways mediated by avian erythroblastosis oncogene B (ErbB) receptors, nuclear factor-kappaB (NF-κB), and the Hedgehog (Hh)/glioma-associated (GLI) oncogene cascade (HH/GLI) plays an important role in neoplastic transformation [3]. Because they are able to modulate the signal transduction pathways involved in carcinogenesis, plant derivatives have promising potential for counteracting tumor growth [3,13,14].

The main drawback to using polyphenols as anticancer agents is their poor bioavailability in the human body, which may hinder their *in vivo* effects, especially when used singly [15,16]. One approach to counteracting this effect may be combination treatment with several polyphenols or with polyphenols and anticancer drugs. This report reviews current knowledge on the anticancer effects of combinations of polyphenols or polyphenols and anticancer drugs, with a focus on their ability to modulate multiple signaling transduction pathways involved in carcinogenesis.

## 2. Classification of Polyphenols

**Figure 1:**
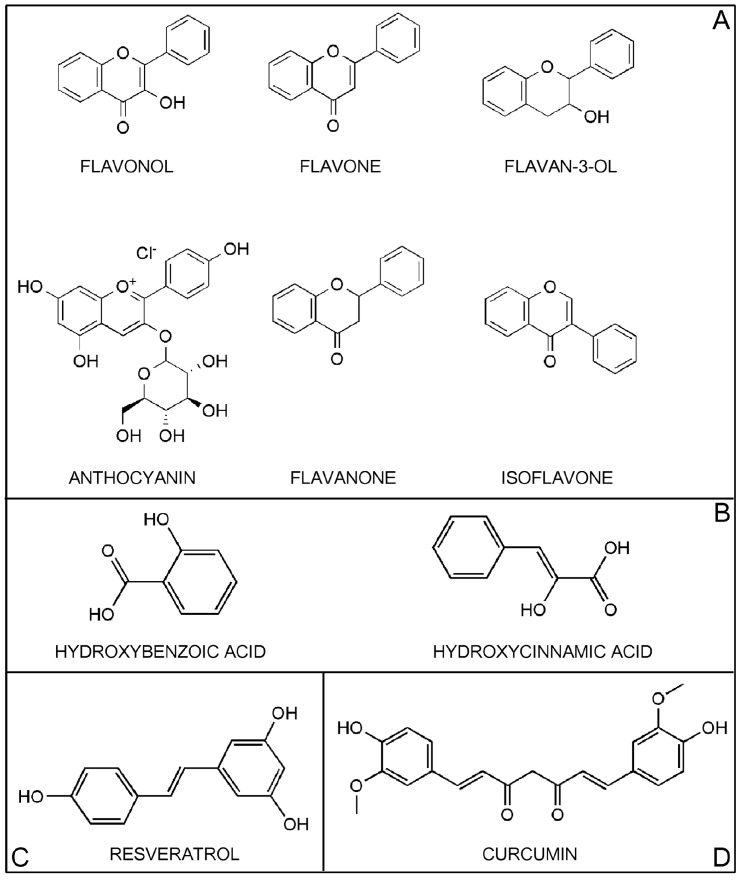
Structure of the major classes of polyphenols. Panel **A**: Flavonoids; Panel **B**: Phenolic acids; Panel **C**: Stilbenes. The figure shows the main member, resveratrol; Panel **D**: Other polyphenols. The figure shows curcumin.

Polyphenols are widely distributed in plant-derived foods. They comprise a large variety of compounds that have a characteristic structure of at least one aromatic ring bearing one or more hydroxyl groups. Polyphenols are classified according to the number of phenol rings that they contain and by the structural elements that bind these rings to one another. The main classes of polyphenols are flavonoids, phenolic acids, stilbenes, and lignans [1,15] (Figure 1).

### 2.1. Flavonoids

Flavonoids, the most abundant polyphenols in our diet, are formed from phenylalanine through a biosynthetic process involving the shikimic acid and acylpolymalonate pathways [17]. Flavonoids consist of 15 carbon atoms with 2 aromatic rings (A- and B-rings) connected by a 3-carbon bridge that binds with 1 oxygen and 2 carbons of the A-ring, forming a third 6-carbon ring (C-ring) [18]. Flavonoids are further classified into subclasses defined by different functional groups and levels of oxidation in the C-ring, and by different connections between the B- and C-rings. Variations between compounds within a subclass consist of different substituents on the A- and B-rings [4] (Figure 1, Panel A).

As well as the different subclasses of flavonoids, worldwide, dietary intake of flavonoids is highly variable. From a dietary standpoint, the most important food-based subclasses of flavonoids are flavonols, flavones, flavan-3-ols, anthocyanins, flavanones, and isoflavones. The flavonoid subclasses dihydroflavonols, flavan-3,4-diols, chalcones, dihydrochalcones, and aurones are minor components of our diet [4].

#### 2.1.1. Flavonols

Flavonols are present in plants in glycosylated form. The sugar component, most commonly glucose or rhamnose, is on the 3-position of the C-ring (Figure 1, Panel A). The main flavonols are quercetin, kaempferol, and myricetin, found mostly in fruits, edible plants, wine, and tea [1]. Although flavonols represent the most abundant flavonoids found in foods, their daily intake is generally low. Several studies have estimated a mean daily intake of 21.4 mg/day (the Netherlands), 22.4 mg/day (Italy), 16.8 mg/day (Denmark), 18.7 mg/day (Spain), 5.4 mg/day (Finland), 19.4 mg/day (Greece), 27.4 mg/day (UK), 16.4 mg/day (Japan), and 12.9 mg/day (USA) [4].

#### 2.1.2. Flavones

The chemical structure of flavones may have a wide range of substitutions, including hydroxylation, methylation, *O*- and *C*-alkylation, and glycosylation. Flavones are present in plants mainly as 7-*O*-glycosides [15] (Figure 1, Panel A). Their most abundant representatives in foods are apigenin (parsley, celery, onion, garlic, pepper, chamomile tea) and luteolin (thai chili, onion leaves, celery). Less abundant flavones include tangeretin and nobiletin (*Citrus* fruits), baicalein and wogonin (*Scutellaria*), and chrysin (*Passiflora*). Estimated daily intake of flavones is very low (0.3–1.6 mg/day) [4].

#### 2.1.3. Flavan-3-Ols

Flavan-3-ols, the most chemically complex subclass of flavonoids, contain a hydroxyl group in the 3-position of the C-ring (Figure 1, Panel A). They exist in monomeric, oligomeric, and polymeric forms and are not glycosylated in foods. The simplest monomers are (+)-catechin and its isomer (−)-epicatechin, whose hydroxylation generates (+)-gallocatechin and (−)-epigallocatechin. (−)-epicatechin-3-*O*-gallate and (−)-epigallocatechin-3-*O*-gallate (EGCG) are formed through an additional esterification with gallic acid in the 3-position of the C-ring. Proanthocyanidins are dimers, oligomers, and polymers of catechins and are subdivided into types A, B, and C. The most common proanthocyanidins found in plants are procyanidins B1, B2, B3, and B4 [1,15]. Flavan-3-ols are found mainly in fruits, berries, cereals, nuts, chocolate, red wine, and tea. Estimated daily intake is very high (12–189.2 mg/day) [4].

#### 2.1.4. Anthocyanins

Anthocyanins are water-soluble pigments mainly present as glycosides of their aglycone form (anthocyanidin) [19] (Figure 1, Panel A). There are more than 550 anthocyanins in nature. They vary according to the number of hydroxyl groups and degree of methylation in the aglycone molecule, the number and position of sugars linked to the aglycone molecule, and the number and nature of aliphatic or aromatic acids linked to these sugars [4]. The most abundant anthocyanins are cyanidin, pelargonidin, delphinidin, peonidin, petunidin, and malvidin. Their main food sources are berries, cherries, red grapes and currants, red wines, blood oranges, the black varieties of soybeans, rice, beans, and the red varieties of onions, potatoes, and cabbage [11]. In the U.S., estimated daily intake of anthocyanins is high compared to other flavonoids (180–215 mg/day) [11].

#### 2.1.5. Flavanones

Flavanones are non-planar flavonoids found mainly in citrus fruits, where they occur mainly as mono- and diglycosides or, less frequently, in aglycone form (Figure 1, Panel A). The most important aglycone flavanones are naringenin and hesperetin. The correspondent glycated forms are neohesperidosides such as naringin (naringenin-7-*O*-neohesperidoside) and neohesperidin (hesperetin-7-*O*-neohesperidoside), and rutinosides, such as narirutin (naringenin-7-*O*-rutinoside) and hesperidin (hesperetin-7-*O*-rutinoside) [15,20]. Hesperetin, naringenin, neohesperidin and naringin are abundant in oranges, grapefruit and tomatoes. The main food sources of hesperidin and narirutin are sweet orange, lemon, mandarin and grapefruit [20]. The estimated mean dietary intake of flavanones is highest in Europe (20.4–50.6 mg/day) and relatively lower in the U.S. (14.4 mg/day) [4].

#### 2.1.6. Isoflavones

Isoflavones are classified as phytoestrogens due to structural similarities with estrogens, particularly 17-ß-estradiol, that confer pseudohormonal activity [18,21] (Figure 1, Panel A). Daidzein, genistein, and glyciten are the most common members of this subclass. They are found mainly in soybeans and soy products, which have the highest levels of isoflavones, and in leguminous plants [22]. In soy products, isoflavones occur as aglycones (genistein and daidzein) or glycosides (genistin and daidzin), depending on how the soy products are processed [22]. Estimated mean dietary intake of isoflavones is very low in Europe and the U.S. (0.1–1.2 mg/day) and higher in Japan and China, where consumption of soy products is more common [4].

#### 2.1.7. Minor Subclass of Flavonoids

Chalcones, dihydrochalcones, aurones, dihydroflavonols, and flavan-3,4-diols make up a minor subclass of flavonoids. The main food sources of chalcones are tomatoes, licorice, shallots, and bean sprouts. Dihydrochalcones (phloretin) occur exclusively in apples and apple products. Aurones (aureusidin) are isomers of flavones that have a limited presence in vegetables and fruits. Dihydroflavonols and flavan-3,4-diols are biosynthetic intermediates of the flavonols and anthocyanins [4].

### 2.2. Phenolic Acids

Phenolic acids are derivatives of benzoic acid and cinnamic acid [1]. Hydroxybenzoic acids have a C_6_-C_1_ structure, are found in few edible plants, and do not have high nutritional value (Figure 1, Panel B). Members of this subclass are protocatechuic acid and gallic acid, the commonest phenolic acid. In its non-sugar galloyl ester form, its main dietary sources are grapes, wine, green and black teas, and mangoes. Gallic acid is also the biosynthetic precursor of hydrolysable tannins (gallotannins and ellagitannins), where it occurs as complex sugar esters. Gallotannins are found in mangoes; ellagitannins occur in red fruits such as strawberries, raspberries, and blackberries [1,15].

Hydroxycinnamic acids have a C_6_-C_3_ structure and are mainly found in glycosylated forms or esters of quinic, shikimic, and tartaric acid (Figure 1, Panel B). The most common hydroxycinnamic acids are caffeic acid, ferulic acid, *p*-coumaric acid, and sinapic acid. When combined, caffeic acid and quinic acid form chlorogenic acid, found in fruits (blueberries, kiwis, plums, cherries, apples) and in high concentrations in coffee (70–350 mg in a single cup) [1]. Caffeic acid, the most abundant phenolic acid, comprises 75%–100% of the total hydroxycinnamic acid content of most fruits. Ferulic acid is the most abundant phenolic acid in cereal grains and comprises up to 90% of the total polyphenol content of wheat grain. Dietary intake of hydroxycinnamic acids is highly variable, depending on coffee consumption [1].

### 2.3. Stilbenes

Stilbenes are phytoalexins (C_6_-C_2_-C_6_ structure) produced by plants as a defense against pathogens, disease, injury, and stress conditions. They have a limited presence in our diet and the main member is resveratrol (3,5,4'-trihydroxystilbene) (RES) [15] (Figure 1, Panel C). RES is present as *cis* and *trans* isomers as well as conjugated derivatives (*trans*-resveratrol-3-*O*-glucoside) in grapes, berries, plums, peanuts, and pine nuts. RES has valuable biological properties, including antioxidant, anti-inflammatory, anticancer, and antiaging activities [23,24,25].

### 2.4. Lignans

Lignans are plant-derived compounds whose structural similarities with estrogens classify them as phytoestrogens, similar to isoflavones [26]. They are formed by oxidation of 2 phenylpropane units and mainly occur prevalently as free form in nature. Lignans are present in high concentration in linseed and in minor concentration in algae, leguminous plants, cereals, vegetables, and fruits [1]. Lignans include ecoisolariciresinol, matairesinol, medioresinol, pinoresinol, and lariciresinol. Human gut microflora metabolize lignans into enterodiol and enterolactone [27].

### 2.5. Other Polyphenols

Among the other polyphenols, Curcumin represents the main compound. Curcumin (CUR) (1,7-bis-(4-hydroxy-3-methoxyphenyl)-1,6-heptadiene-3,5-dione), a member of the curcuminoid family, is a polyphenol compound found in turmeric, a spice produced from the rhizome of *Curcuma longa* [28] (Figure 1, Panel D). CUR has been studied extensively in recent years as a pleiotropic molecule able to interact with a variety of molecular targets and signal transduction pathways. It has been found to have antitumor, anti-inflammatory, antioxidant, immunomodulatory, and antimicrobial activities in both rodents and humans. CUR is considered a “multifunctional drug” due to its ability to modulate the activity of multiple targets involved in carcinogenesis through direct interaction with gene expression [13,29].

## 3. Polyphenols Target Signal Transduction Pathways Involved in Carcinogenesis

As stated earlier, signal transduction pathways involved in carcinogenesis often interact with each other, enhancing oncogenic signals that trigger the malignant phenotype of cells [3,10]. For example, cross-talk between the signaling pathways mediated by ErbB receptors, NF-κB, and the HH/GLI cascade plays an important role in neoplastic transformation [3].

Polyphenols can inhibit cancer cell growth by interacting with multiple signaling pathways. In this regard, different studies reported the ability of polyphenols to modulate ErbB receptors, HH/GLI and NF-κB signaling pathways in cancer cells both *in vitro* and *in vivo*.

### 3.1. Modulation of ErbB Receptors Signaling Pathway by Polyphenols in Cancer Cells

ErbB family receptors include epidermal growth factor receptor (EGFR), ErbB2 (Neu, HER2), ErbB3, and ErbB4, each of which is involved in carcinogenesis. It has been demonstrated that, after binding with specific ligands, dimerization and receptor trans-phosphorylation of ErbB receptors occur [30]. This phenomenon activates several downstream molecules that in turn activate the mitogen-activated protein kinase (MAPK) pathway, leading to increased cell proliferation and differentiation [3,31].

Polyphenols such as CUR, EGCG, RES, quercetin and apigenin have demonstrated a potent activity in affecting ErbB receptors downstream signaling in several types of cancer cells.

Squires *et al.* found that, in the MDA-MB-468 breast cancer cell line, CUR suppressed EGFR phosphorylation, inhibited *c-fos* expression and ERK activity *in vitro* [32]. CUR has also been shown to (a) decrease the expression of phosphorylated forms of EGFR and ERK1/2; (b) induce apoptosis and cell cycle arrest; and (c) inhibit cell proliferation in the aggressive MDA-MB-231 breast cancer cell line *in vitro* [33,34].

It also been demonstrated that CUR induced apoptosis in several breast cancer cell lines and delayed the growth of mammary tumors in BALB-*neu*T transgenic mice. *In vitro* experiments showed that CUR inhibited the growth of breast cancer cell lines in a dose-dependent manner by enhancing the activation of apoptosis and down-regulating the activity of ERK1/2 MAPKs. In addition, the cytotoxic effects of CUR were observed in breast cancer cells expressing either high or low levels of ErbB2/neu. These results were confirmed by *in vivo* experiments. BALB-*neu*T transgenic mice were treated with CUR starting at the age at which they displayed atypical breast hyperplasia (6 weeks) or invasive breast carcinoma (16 weeks). CUR administration resulted in a significant reduction of tumor multiplicity in both early and in an advanced stage of mammary carcinogenesis, and did not modify hematological and clinical chemistry parameters in all treated mice [29].

Similar effects were also reported in gastrointestinal, prostate, pancreatic, lung, and ovarian cancer cells. Cai *et al.* showed that CUR demonstrated a block of proliferation and invasion of gastric cancer cells by inhibiting the expression of ErbB2 and cyclin D1 and suppressing the activity of p21-activated kinase 1 (PAK1), a downstream protein of EGFR [35].

Furthermore, in androgen responsive and refractory prostate cancer cells *in vitro*, CUR inhibited cell proliferation by down-regulating EGFR and ErbB2 expression [36]. CUR has also proved to be effective in inhibiting proliferation and inducing apoptosis of pancreatic and lung adenocarcinoma cells, through the modulation of cyclooxygenase-2 (COX-2), EGFR and phospho-ERK1/2 expression [37].

In HEY ovarian cancer cell line, CUR induced apoptosis in a p53-indipendent manner through the activation of p38 kinase, the down-regulation of Bcl-2 and surviving expression and the modulation of Akt signaling [38].

Pan *et al.* evaluated the ability of EGCG to counteract the growth of ErbB2- or/and ErbB3-overexpressing breast cancer cells. They reported that the anticancer activity of this polyphenol should be due to its peculiarity to interfere with heterodimerization and tyrosine phosphorylation of ErbB2-ErbB3, leading to an inhibition of the MAPK cascade pathway [39]. In an *in vitro* study on ErbB2/*neu-*overexpressing mouse mammary tumor NF639 and SMF cells, Pianetti *et al.* showed that EGCG decreased the phosphorylation and constitutive activation of ErbB2/*neu*, and suppressed the MAPK and NF-κB pathways, determining a sensible reduction of tumor growth [40]. Moreover, EGCG has been proved to have inhibitory properties on cell proliferation of breast and head and neck squamous cell carcinomas (HNSCC) by suppressing the activity of EGFR, signal transducer and activator of transcription 3 (STAT3), Akt and *c-fos* [41,42]. Similar effects have been observed in human colon and non-small cell lung cancer (NSCLC) cells. EGCG has demonstrated a potent ability in modulating EGFR signaling in several colon cancer cell lines [43,44,45,46]. In particular, it has been observed that EGCG was able to reduce the cellular levels of EGFR, ErbB2 and ErbB3 in the SW837 colon carcinoma cell line [43].

RES has been shown to be effective in inducing growth inhibition and apoptosis through the modulation of MAPK signaling in different cancer cell lines. In liver HepG2 cancer cell line, RES induced a suppression of cell proliferation and enhanced apoptosis by down-regulating cyclin D1, Akt, p38 kinase and Pak 1 expression, and increasing phospho-ERK1/2 protein levels [47]. Similar effects were also observed in epidermoid carcinoma and colon cancer cells [48,49].

The modulatory activity of quercetin on ErbB receptors signaling was investigated in several cancer cell lines. Jeong *et al.* reported that the plant polyphenol quercetin inhibited ErbB2 signaling pathway in breast cancer cells. In particular, in SKBR3 breast cancer cell line, quercetin treatment resulted in a suppression of tyrosine kinase activity and in a reduction of ErbB2/neu protein level as well as in a dephosphorylation of phosphatidylinositol-3-kinase (PI3K) and Akt [50].

Quercetin exerted its modulatory activity also on hepatoma and lung cancer cells. In the human hepatoma HepG2 cell line quercetin led to cell death by inducing suppression of Akt and ERK1/2 phosphorylation and modulating the NF-κB pathway [51]. Similarly, in A549 lung cancer cells, quercetin induced growth inhibition and apoptosis, in a dose-dependent manner, through the inactivation of Akt-1 and the increase of ERK-MEK1/2 phosphorylation [52].

The anticancer effects of the flavonoid apigenin have been evaluated in different types of cancer cells. In PC-3 and LNCaP prostate cancer cell lines, apigenin suppressed cell proliferation, induced an arrest of the cell cycle in G0/G1 phase and reduced the phosphorylation of Rb, p38 kinase and *c-fos* protein [53]. Moreover, in breast cancer cells, at high doses, apigenin resulted in an arrest of cell growth by inhibiting the activity of several kinases involved in the downstream signaling following EGFR activation [54]. In another study performed on HNSCC cells *in vitro*, apigenin treatment inhibited survival and induced apotosis by reducing ligand-induced phosphorylation of EGFR and ErbB2 and modulating their downstream signaling [55].

Finally, Lee *et al.* evaluated the capacity of polyphenols extracted from *Allium cepa* Linn (PEAL) to modulate the MAPK pathway in AGS human gastric carcinoma cell line. They found that PEAL arrested tumor growth and induced apoptosis in a dose-dependent manner. This feature was associated with their ability to up-regulate p53 expression, increase Bax/Bcl-2 ratio and block Akt activity [56]. Effects of polyphenols on ErbB receptors signaling pathway in cancer cells are summarized in Table 1.

**Table 1:** Modulation of ErbB receptors, NF-κB and HH/GLI signaling pathways by polyphenols in cancer cells.

Signaling Pathway	Treatment	*In Vitro* Model	*In Vivo* Model	Antitumoral Effects	Reference
ErbB receptors	CUR	MDA-MB-468 breast cancer cells (40 µM)		↓ EGFR phosphorylation ↓ *c-fos* expression ↓ ERK, MKK4, JNK activity	[32]
		MDA-MB-231 breast cancer cells (30–50 µM)		↓ Cell proliferation ↓ EGFR, ERK1/2, Akt, MAPK phosphorylation	[33,34]
		Breast cancer cells (6–50 µM)	BALB-*neu*T transgenic mice (2 mg in 50 µL corn oil p.o. thrice weekly)	↓ Tumor growth ↓ ERK1/2 activity ↑ Bax/Bcl-2 ratio ↑ PARP cleavage ↓ Tumor multiplicity	[29]
		Gastric cancer cells (1–100 µM)		↓ Cell proliferation ↓ ErbB2, cyclin D1 expression ↓ PAK1 activity	[35]
		LNCaP, C4-2B prostate cancer cells (0–100 µM)		↓ Cell proliferation ↓ EGFR, ErbB2 expression	[36]
		Pancreatic and lung cancer cells (0–50 µM)		↓ Cell proliferation ↓ COX-2, EGFR, phospho- ERK1/2 expression	[37]
		HEY ovarian cancer cells (2.5–160 µM)		↓ Bcl-2, Akt expression ↑ p38 activity	[38]
ErbB receptors	EGCG	MCF-7 breast cancer cells (5–20 µM)		↓ ErbB2, ErbB3 phosphorylation ↓ MAPK pathway	[39]
		mammary tumor NF639 and SMF cells (0–80 µg/mL)		↓ Cell proliferation ↓ ErbB2/*neu* phosphorylation ↓ NF-κB, MAPK pathways	[40]
		HNSCC (10 µg/mL), breast cancer cells (30 µg/mL)		↓ Cell proliferation ↓ EGFR, STAT3, Akt, *c-fos* activity	[41,42]
		SW837 colon carcinoma cells (30 µg/mL)		↓ EGFR, ErbB2 and ErbB3 cellular levels	[43]
	RES	HepG2 liver cancer cells (50–300 µM)		↓ Cell proliferation ↓ Cyclin D1, Akt, p38 kinase expression ↑ Phospho-ERK1/2 protein levels	[47]
		A431 epidermoid carcinoma cells (0–100 µM)		↓ Cyclin D1, MEK1, ERK1/2 expression	[48]
		HT-29 colon cancer cells (25 µM)		↓ JACK-STAT pathway ↓ iNOS, COX-2 expression	[49]
	Quercetin	SKBR3 breast cancer cells (100–200 µM)		↓ ErbB2 tyrosin kinase activity ↓ PI3K, Akt phosphorylation	[50]
		HepG2 liver cancer cells (50 µM)		↓ ERK1/2, Akt phosphorylation ↓ NF-κB pathway	[51]
		A549 lung cancer cells (0–58 µM)		↓ Cell proliferation ↓ Akt-1 activation ↑ ERK-MEK1/2 phosphorylation	[52]
	Apigenin	PC-3, LNCaP prostate cancer cells (5–40 µM)		↓ Cell proliferation ↑ Proportion of cells in G0/G1–phase ↓ Rb, p38 kinase and *c-fos* phosphorylation	[53]
		HNSCC cells (6–100 µM)		↓ Cell proliferation ↓ EGFR, ErbB2 phosphorylation	[55]
NF-κB	EGCG	A431 epidermoid carcinoma cells (10–40 µg/mL)		↓ Cell proliferation ↓ NF-κB/p65 nuclear translocation	[57]
	Delphinidin	PC-3 prostate cancer cells (30–180 µM)	Athymic (nu/nu) nude mice bearing prostate cancer tumors (2 mg i.p. thrice weekly)	↓ Tumor growth ↓ IκB kinase γ , IκB-α phosphorylation ↓ NF-κB DNA binding activity	[58,59]
		HCT-116 colon cancer cells (30–240 µM)		↓ Cell proliferation ↓ IκB-α phosphorylation ↓ NF-κB activation	[60]
	Anthocyanin		rats with esophagus tumor (3.8 μmol/g/day p.o.)	↓ Tumor development ↓ NF-κB, COX-2 expression	[61]
		CAL-27 oral cancer cells (0–500 µg/mL)		↓ Cell proliferation, metastasis ↓ NF-κB, MMPs expression ↓ MAPK pathway	[62]
	CA, CAPE	HepG2 liver cancer cells (CA 100 µg/mL; CAPE 5 µg/mL)	nude mice injected with HepG2 cells (CA + CAPE 5 mg/kg s.c thrice weekly; CA + CAPE 20 mg/kg/day p.o. for 5 weeks)	↓ Tumor growth ↓ NF-κB, MMP-9 activity ↓ Liver metastasis	[63]
	CUR	Cervical cancer cells (5–60 µM)		↓ IκB-α phosphorylation ↓ NF-κB activation	[64]
			ICR mice (1–25 µM)	↓ COX-2 expression ↓ NF-κB activation ↓ NF-κB nuclear translocation ↓ ERK1/2 activity	[65]
NF-κB	RES	MCF-7 breast cancer cells (50–150 µM)		↓ Cell proliferation ↓ NF-κB activation ↓ Bcl-2 expression	[66]
		OCIM2, OCI/AML3 myeloid leukemia cells (5–75 µM)		↓ Cell proliferation ↓ NF-κB activation ↑ PARP cleavage ↑ Proportion of cells in S-phase	[67]
HH/GLI	CUR	medulloblastoma cancer cells (40 µM)		↓ SHH, GLI1, PTCH1 expression ↑ Proportion of cells in G2/M-phase	[68]
	EGCG	SW1353, CRL-7891 chondrosarcoma cells (0–4 µM)		↓ Cell proliferation ↓ GLI1, PTCH1 expression	[69]
		pancreatic cancer stem cells (20–60 µM)		↓ Cell proliferation, invasion ↓ SMO, PTCH1, PTCH2, GLI1, GLI2 expression	[70]
	Apigein, baicalein, CUR, RES EGCG, genistein, quercetin	Pancreatic cancer stem cells, prostate cancer cells (20–30 µM)		↓ GLI1 expression	[71,72]

Abbreviations: p.o., per os; i.p., intraperitoneally; i.t., intratumorally; i.v., intravenously; s.c., subcutaneously.

### 3.2. Modulation of the NF-κB Pathway by Polyphenols in Cancer Cells

Constant activation of the NF-κB-mediated pathway is another feature of tumor cells. NF-κB is a transcription factor retained in an active state in the cytoplasm by its inhibitors, the IκB proteins (IκBs). The IκB proteins bind to NF-κB, masking its nuclear localization signals and preventing its nuclear translocation [73]. Activation of the IκB kinase (IKK) complex promotes phosphorylation of the IκBs and their consequent proteasomal degradation. Degradation of IκBs promotes NF-κB nuclear translocation. Once in the nucleus, NF-κB activates the transcription of several genes involved in inflammation, cell growth, and invasivity [74]. Constant activation of NF-κB in cancer cells is linked to high production of inflammatory mediators such as tumor necrosis factor (TNF), interleukin-1 (IL-1), IL-6, prostaglandin E2 (PGE2), and reactive oxygen species (ROS) within the tumor microenvironment [3,75,76]. Up-regulation of NF-κB activity is also involved in the development of chemoresistance in tumor cells, which leads to inhibition of apoptosis, increased angiogenesis, and metastatic capability [77].

Gupta *et al.* demonstrated that EGCG inhibited cell proliferation and induced apoptosis in a dose-dependent manner by reducing nuclear translocation of NF-κB/p65 in human epidermoid carcinoma A431 cells [57]. Similar effects on tumor growth of different cancer cells were achieved *in vitro* and *in vivo*, using anthocyanins.

Hafeez *et al.* observed that delphinidin induced cell growth arrest and caspase-dependent apoptosis in a dose-dependent manner in prostate cancer cells *in vitro*, by reducing phosphorylation of IκB kinase γ and of NF-κB inhibitory protein IκB-α, and inhibiting NF-κB DNA binding activity. Moreover, *in vivo*, administration of delphinidin resulted in a significant decrease of tumor growth and NF-κB protein levels in mice bearing prostate cancer tumors [58,59].

Delphinidin exerted similar effects also in human colon cancer cells. Indeed, HCT-116 colon cancer cells treated with delphinidin showed a strong reduction of proliferation and induction of apoptosis. This phenomenon was due to the ability of this flavonoid to inhibit the activation of IKKα and IκB-α phosphorylation and the constitutive activation of NF-κB [60].

In addition, Wang *et al.* concluded that a diet containing anthocyanins from black raspberries inhibited the development of nitosomethylbenzylamine (NMBA)-induced rat esophagus tumors by reducing expression of NF-κB and COX-2 at tumor level [61].

In another study, Fan *et al.* observed that anthocyanins from black rice blocked oral cancer CAL-27 cells metastasis *in vitro* by decreasing metalloproteinases (MMPs) and NF-κB expression, and suppressing the MAPK pathway [62].

Caffeic acid (CA) and its derivative caffeic acid phenethyl ester (CAPE) were found to possess anticancer and antimetastatic activities on HepG2 hepatocarcinoma cells. These two compounds suppressed tumor growth both *in vitro* and *in vivo* by inhibiting NF-κB and metalloproteinase-9 (MMP-9) activity. In addition, *in vivo*, the subcutaneous and oral administrations of CA and CAPE led to a significant reduction of the liver metastasis of HepG2 tumor xenografts in nude mice [63]. CUR and RES were shown to modulate the NF-κB pathway as well.

CUR was able to induce apoptosis, suppress IκB-α phosphorylation and enhance the inhibition of NF-κB activation in cervical cancer cells [64].

In an *in vivo* study, employing a mouse skin tumor model, Chun *et al.* reported that CUR exerted its antitumor effects by suppression of COX-2 protein expression, activation of NF-κB and its nuclear translocation, and inhibition of the catalytic activity of ERK1/2 in mouse skin [65].

RES has been shown to counteract tumor growth of MCF-7 breast cancer cell line *in vitro*, by down-regulating Bcl-2 expression and suppressing NF-κB activity [66].

Furthermore, the inhibition of IL-1β-induced activation of NF-κB by RES determined suppression of cell growth, enhancement of apoptosis and induction of cell cycle arrest in S-phase in acute myeloid leukemia cells [67].

Effects of polyphenols on the NF-κB signaling pathway in cancer cells are summarized in Table 1.

### 3.3. Modulation of HH/GLI Pathway by Polyphenols in Cancer Cells

The HH/GLI cascade is a complex signaling transduction pathway that controls cell proliferation, survival, and differentiation in vertebrate embryogenesis. Activation of HH signaling promotes translocation of the trans-membrane protein, called smoothened (SMO), in the “primary cilium”, a non-motile structure protruding from the cell surface. Once SMO enters the cilium, it promotes the activation of cytoplasmic GLI proteins and their translocation to the nucleus, leading to the transcription of HH target gene products. Aberrant HH signaling has been implicated in the development and/or progression of several cancer types, including basal cell carcinoma (BCC), rhabdomyosarcoma, and gastrointestinal, lung, breast, and brain tumors [3,78,79,80].

Among polyphenols, CUR, EGCG and genistein exerted a potent modulatory activity on HH/GLI pathway in cancer cells. In medulloblastoma cells, CUR was able to arrest cells at the G2/M phase of the cell cycle and induce apoptosis by down-regulating the expression of the sonic hedgehog homolog (SHH) protein and its downstream molecules GLI1 and protein patched homolog 1 (PTCH1) [68]. A recent study by Tang *et al.* showed that EGCG affected proliferation and promoted apoptosis of SW1353 and CRL-7891 human chondrosarcoma cells in a dose-dependent manner by inhibiting PTCH1 and GLI protein expression [69].

EGCG treatment resulted in a suppression of cell proliferation and invasion of pancreatic cancer stem cells. This polyphenol exerted its anticancer activity by suppressing SMO, PTCH1, PTCH2, GLI1 and GLI2 expression [70]. In the same cells, a modulatory activity on HH/GLI pathway was also displayed by genistein [71].

Finally, Slusarz *et al.* reported that, when administered individually, apigein, baicalein, CUR, EGCG, genistein, quercetin, and RES inhibited the growth of prostate cancer cells *in vitro* and *in vivo*, through modulation of the HH signaling pathway [72].

Effects of polyphenols on the HH/GLI signaling pathway in cancer cells are summarized in Table 1.

### 3.4. Cross-Talk between ErbB Receptors and the HH/GLI and NF-κB Signaling Pathways in Cancer Cells

Cross-talk between ErbB receptors and the HH/GLI and NF-κB signaling pathways has been shown to promote the transformation and proliferation of cancer cells. For example, the EGFR and MAPK signaling pathways promote the nuclear localization and transcriptional activity of GLI1 protein in melanoma and other cancer cells [81,82,83,84,85]. It was also reported that IKKα induces ErbB2 to activate NF-κB through the canonical pathway by enhancing the invasive capacity of ErbB2^+^ breast cancer cells [86]. In addition, studies have shown that NF-κB promotes ErbB2-mediated tumorigenesis *in vivo* by enhancing tumor neo-angiogenesis, and that IKKα plays an important role in providing stimuli that maintain mammary tumor-initiating cells [87,88]. Makino *et al.* reported that up-regulation of IKKα and IKKß by the integrin-linked kinase/Akt pathway promotes the ErbB2-mediated NF-κB anti-apoptotic pathway [89].

### 3.5. Other Signal Transduction Pathways Involved in Carcinogenesis

As described in the following sections, in addition to signal transduction pathways mentioned above, polyphenols are able to modulate other signals involved in carcinogenesis, such as cell cycle, apoptosis, and angiogenesis.

Two events strictly associated with carcinogenesis are the loss of function of cell cycle checkpoint genes and the inhibition of the apoptotic process.

Cell cycle checkpoints genes control cell cycle progression and ensure a high regulation of crucial events such as DNA replication and chromosome segregation. In normal cells, when damage to DNA occurs, checkpoints genes respond to damage by temporarily arresting the cell cycle by enhancing transcription of genes that facilitate repair, in order to avoid that the DNA lesion is transmitted to daughter cells during the mitosis. If the DNA damage is not repaired, cell cycle is completely arrested and the apoptotic process is activated. Loss of function of checkpoint genes results in genomic instability and has been related in the transformation of normal cells into cancer cells [9,90].

One of the most important genes involved in the regulation of the cell cycle is p53, because it plays a central role in eliminating the genomic damage and in inducing the apoptotic process. It is not surprising that over 70% of human cancers have mutations that lead to a loss of function of this gene or an inhibition of its downstream signal transduction pathway [91]. In addition, the frequent loss of p53 function seems to be related to the acquisition of cross-resistance to anticancer agents in human tumors [92,93,94].

It has been also reported that in addition to inhibiting p53 function by mutation, other p53 independent mechanisms are utilized by many cancers to alter the apoptotic process. For example, the overexpression of the anti-apoptotic bcl-2 family members (Bcl-2, Bcl-xL, MCL1, Bcl-W, A1, and Bcl-B), the suppression of activation of pro-apoptotic members (Bax, BaK, and Bok), the aberrant expression of inhibitors of apoptosis (IAP) proteins and cellular FLICE-like inhibitory protein (C-FLIP) have been found to be related to cancer cells resistance to apoptotic stimuli and have been associated to the progression of several types of tumors [95].

Angiogenesis is a crucial process required to maintain the growth and persistence of primary tumors and to enhance the metastatic dissemination. Indeed, increased vascularization seems to be involved with the invasive properties of tumors, leading to a more aggressive malignant tumor phenotype [96].

Tumor cells induce neo-angiogenesis and enhance expression of pro-angiogenic factors such as vascular endothelial growth factor (VEGF) and fibroblast growth factors (FGF-1 and -2) [97].

PGE2 can also stimulate production of pro-angiogenic factors, activate the MAPK pathway, and increase the transcriptional activity of NF-κB [3].

In summary, the capacity of polyphenols to interact with different pathways involved in carcinogenesis makes them multi-targeting agents with the potential for clinical benefit when employed in cancer treatment [3,6,7,13,14,29,98,99,100].

## 4. Bioavailability of Polyphenols

An important topic of recent research is the analysis of the bioavailability of polyphenols. Epidemiological studies have shown an association between a diet rich in polyphenols and the prevention of human diseases. However, such beneficial effects are dependent on the proportion of active substances that are absorbed from the gastrointestinal tract [15,16]. Indeed, some of the most abundant polyphenols in our diet have little or no beneficial effect because of the low bioavailability of their bioactive metabolites [1]. In order to produce effects *in vivo* a compound must enter the circulation and reach the tissues, in its native or metabolized form, in a sufficient quantity to exert biological activity. Bioavailability is the quantity of a compound that is absorbed and metabolized in the human body after it is ingested, and is commonly measured in terms of maximum plasma concentration (C_max_) [4]. Polyphenols with the best bioavailability are gallic acid and isoflavones, followed by caffeic acid, flavanones, catechin, and quercetin glucosides. Anthocyanins and proanthocyanidins have the lowest bioavailability [101].

Polyphenols occur in various chemical structures that influence their intestinal absorption and the kinds of metabolites circulating in plasma [102]. In their native forms, aglycone polyphenols can be absorbed by the small intestine through passive diffusion, reaching immediately the C_max_. In contrast, polyphenols in the form of esters, glycosides, or polymers undergo an intense metabolism in the small intestine before being absorbed. Glycosilated polyphenols, such as flavonols, isoflavones, flavones and anthocyanins have to be first hydrolyzed by intestinal enzymes (glucosidases and lactase-phlorizin hydrolase) or colon microflora prior to absorption. Glycosylation influences chemical and biological properties of the polyphenols, because removal of the hydrophilic moiety is usually necessary to these polyphenols to pass the intestine membrane by passive diffusion. Glycosylated polyphenols are usually absorbed more slowly than aglycone polyphenols in the intestine [1,102].

Moreover, after hydrolyzation, these polyphenols derivatives are then further metabolized in both the small intestine and the liver. They undergo a conjugation by methylation, sulfation, or glucuronidation before entering circulating plasma and target tissues. In this regard, metabolites that reach plasma are chemically different from polyphenols present in edible foods and this fact could dramatically alter their biological properties [102]. Finally, polyphenols are secreted in bile or eliminated in urine [1].

In summary, several mechanisms limit the bioavailability of polyphenols, including their metabolism in the gastrointestinal tract and liver, their binding on the surfaces of blood cells and microbial flora in the oral cavity and gut, and regulatory mechanisms that prevent the toxic effects of high flavonoid levels on mitochondria or other organelles [103].

The presence of certain types of metabolites in the plasma could depend on the interaction of polyphenols with colonic microflora. Changes in the composition of the colonic microflora could be on the basis of the inter-individual variations in bioavailability of several polyphenols. A better knowledge of the proportions of the plasma phenolic metabolites absorbed by the small intestine or by the colon after transformation by microflora is necessary to well elucidate mechanisms that influence the bioavailability of polyphenols [102].

In addition to endogenous factors, dietary factors can affect the bioavailability of polyphenols. A particular food matrix can affect the release of polyphenols, and food preparation techniques can alter the composition and structure of polyphenols [104]. For all these reasons, only nano- or micromolar concentrations of polyphenols and polyphenol metabolites are found in plasma (0–4 µM after an intake of 50 mg of aglycone equivalents) [101]. Even long-term consumption of flavonoid-rich foods appears insufficient to overcome this problem [103]. Thus, the main drawbacks to the use of polyphenols as single therapy are inadequacies of absorption, biodistribution, metabolism, and bioavailability in the human body.

Compositions and methods for enhancing flavonoids bioavailability, solubility and stability in the human body have been patented, creating new flavonoids derivatives with a better biological activity and stability [4]. In addition, a promising strategy for improving the anticancer effects of polyphenols *in vivo* is the combined use of several polyphenols, or the combination of polyphenols and conventional cancer treatments such as chemotherapy, radiotherapy, and biopharmaceuticals.

## 5. Combinations of Polyphenols: *In Vitro* and *in Vivo* Antitumoral Effects

Several *in vitro* and *in vivo* studies have shown that treatment with polyphenols in combination is more effective in inhibiting cancer growth than treatment with a single polyphenol.

The association between pterostilbene and quercetin, two structurally related small polyphenols, has been investigated in a preclinical study, using the highly malignant B16 melanoma F10 cell line (B16M-F10). *In vitro* experiments showed that these two polyphenols worked synergistically to accumulate cancer cells in the G0/G1 phase and to inhibit metastasis. These findings were confirmed by *in vivo* experiments. Mice injected in the spleen with B16M-F10 cells, and then treated with an intravenous infusion of 20 mg/kg/day of quercetin and pterostilbene, showed a strong inhibition of the metastasis of melanoma cells to the liver and a better host survival compared to treatment with a single polyphenol [105].

Sakamoto demonstrated that the combined use of the isoflavone genistein and the polyphenol thearubigin, found in black tea, inhibited prostate cancer cell growth *in vitro*. Thearubigin, that alone did not inhibit growth in the human prostate tumor cell line PC-3, when combined with genistein at a 1:40 ratio, synergistically inhibited cell proliferation and induced a G2/M phase cycle arrest in a dose-dependent manner [106].

In another *in vivo* experiment, the combination of genistein and RES acted as chemoprevention against prostate cancer in SV-40 Tag rats. Rats fed a diet with high levels of genistein and RES (250 mg/kg) showed an 11.5-fold decrease in prostate cancer incidence compared to controls. These effects were due to the capacity of genistein and RES to inhibit cell proliferation, decrease expression of insulin-like growth factor-1 (IGF-1) protein, and modulate sex steroid receptor and growth factor signaling in the prostate [107].

Wang *et al.* showed that the combination of quercetin and the flavonoid EGCG, found in green tea, decreased proliferation of human prostate cancer cells *in vitro* and *in vivo*. Quercetin enhanced the antiproliferative activity of EGCG in androgen-independent PC-3 cells and in androgen-dependent LNCaP prostate cancer cells *in vitro* by increasing the intracellular concentration of EGCG. The combined treatment caused cell cycle arrest and induced apoptosis in PC-3 cells. Quercetin also had an additive effect in LNCaP cells when administered in combination with EGCG [108].

Similarly, combined treatment with quercetin and EGCG reduced the growth of androgen-sensitive LAPC-4 prostate cancer cells more efficiently than treatment with either compound alone when injected subcutaneously in severe combined immunodeficiency (SCID) mice. Moreover, the combined treatment significantly inhibited tumor-cell proliferation, androgen-receptor expression, and PIK3/Akt signaling, and induced apoptosis *in vivo*. These results support the possibility to use these nontoxic compounds in combination as chemoprevention for cancer patients [109].

Another combination of polyphenols, that of EGCG and CUR, strongly suppressed the growth of NSCLC cells and breast cancer cells both *in vitro* and *in vivo* [110,111]. Zhou *et al.* found that, at low concentrations, the combination of EGCG and CUR synergistically enhanced cell cycle arrest of the NSCLC cell lines A549 and NCI-460 by blocking cells in the G1 and S/G2 phases. *In vivo*, the combination of EGCG and CUR strongly suppressed tumor growth, with no toxicity, in a lung cancer xenograft nude mouse model, suggesting that the combination of these two polyphenols may prevent NSCLC in humans [110].

Similarly, Somers-Edgar *et al.* demonstrated that the combination of EGCG and CUR suppressed breast cancer cell growth *in vitro* and *in vivo*. EGCG plus CUR had a synergic cytotoxic effect on the human breast cancer cell line MDA-MB-231, an effect that correlated with G2/M-phase cell cycle arrest. In addition, female athymic nude mice, implanted with MDA-MB-231 cells and treated with CUR (200 mg/kg/day) and EGCG (25 mg/kg/day) for 10 weeks, showed greater reduction of tumor volume compared to EGCG-, CUR- and vehicle control-treated mice. Moreover, this study also indicated that the combined treatment allowed for a reduced dose of CUR to achieve tumor suppression, suggesting the potential use of these two polyphenols in combination to treat breast cancer in humans [111].

Wang *et al.* reported that adding arctigenin (Arc), a novel anti-inflammatory lignan obtained from *Arctium lappa* seeds, to CUR and EGCG, synergistically increased the chemopreventive effect in the LNCaP prostate cancer cell line and the MCF-7 breast cancer cell line, compared to treatment with CUR, EGCG, or Arc alone. In particular, both Arc and EGCG enhanced CUR’s ability to induce apoptosis in LNCaP cells; in MCF-7 cells, this effect was induced only by the combination of Arc and CUR. In both cell lines, the combined treatment reduced NF-κB, PI3K/Akt, and STAT3 expression and inhibited cell migration compared to any of the compounds used alone, suggesting a promising potential for the use of these three compounds in combination in clinical practice [112].

Amin *et al.* evaluated the anticancer effect of EGCG plus luteolin on head and neck, and lung cancer cell lines. At low doses, the combination synergistically increased apoptosis in both cell lines by activating mitochondrial-dependent and -independent processes. This combination also had an inhibiting effect on tumor growth in experiments with mice implanted with head and neck and lung cancers. In particular, a significant decrease in Ki-67 expression and an increase in TUNEL^+^ cells were observed in tissue from xenograft models [113].

Polyphenol combinations have also shown promising effects in the treatment of leukemia. Working with MOLT-4 human leukemia cells, Mertens-Talcott *et al.* demonstrated that ellagic acid significantly and synergistically enhanced the ability of low-concentration quercetin to reduce cell proliferation, alter the cell cycle, and induce apoptosis [114]. In addition, ellagic acid and quercetin worked synergistically with RES to induce apoptosis and inhibit the growth of MOLT-4 cells. Finally, the combinations of ellagic acid plus RES and quercetin plus RES worked synergistically to induce caspase-3 activity, demonstrating again that the combination of several polyphenols has greater anticancer activity than a single polyphenol used alone [115].

The combined use of polyphenols has shown promise as a strategy against malignancies with poor prognoses, such as neuroblastoma, rhabdomyosarcoma, osteosarcoma, and head and neck carcinomas [116,117,118,119]. Liontas *et al.* investigated the ability of CUR and RES combined to induce apoptosis and nuclear translocation, and to activate p53 in human neuroblastoma, an aggressive childhood cancer of the peripheral nervous system that has a poor prognosis. The combination of CUR and RES decreased cell proliferation in a dose- and time-dependent manner, and induced cell cycle arrest and apotosis in several neuroblastoma cell lines *in vitro*. The combination also produced a transient up-regulation of p53 expression and induced nuclear translocation as well as p21 and Bax expression. The potential anticancer effect of these two polyphenols in combination may represent a new strategy for treating advanced-stage or chemo-resistant neuroblastoma [116].

The anticancer activity of the combination of diallyldisulfide (DADS), RES, and CUR has also been evaluated in malignant tumors of mesenchimal origin, such as rhabdomyosarcoma and osteosarcoma, both of which are highly aggressive pediatric malignancies with poor prognoses. The combination treatments of DADS plus RES, DADS plus CUR, and RES plus CUR were compared to treatment with single compounds. Results showed that, compared to single compounds, the combination treatments had greater *in vitro* anticancer activity on malignant rhabdoid (SJ-RH4, RD/18) or osteosarcoma (Saos-2) cell lines. In particular, RES and DADS potentiated the apoptotic effects of CUR on SJ-RH4 and RD/18 cell lines, suggesting that CUR-based combinations may have relevance for the treatment of p53-deficient tumor cells, which are often unaffected by conventional chemotherapies or radiotherapy [117].

RES also potentiated the *in vitro* and *in vivo* anticancer effects of CUR in HNSCC. A study reported that, compared to CUR alone, the combination of RES plus CUR increased PARP-1 cleavage, the Bax/Bcl-2 ratio, inhibition of ERK1 and ERK2 phosphorylation, and expression of the autophagic marker LC3 II in HNSCC cell lines. The model of compounds interaction indicated the onset of an additive effect of the two compounds compared to the single treatment after decrease of their concentrations. In addition, treatment with RES plus CUR reduced the growth of transplanted salivary gland cancer cells in BALB/c mice more efficiently than either CUR or RES alone [118]. Similar effects were seen in a study by Elattar *et al.*, who found that RES combined with quercetin significantly increased inhibition of cell growth and DNA synthesis compared to quercetin alone in the SCC-25 oral squamous carcinoma cell line [119].

Finally, the combination of CUR and RES inhibited the growth of p53^+^ (wild type) and p53^−^HCT-116 colon cancer cells *in vitro* and *in vivo* more effectively than either of the compounds used alone. Furthermore, compared to single compounds, CUR plus RES synergistically inhibited cell proliferation, stimulated apoptosis, attenuated NF-κB activity, and inhibited activation of EGFR and its family members. These results suggest that the combination of CUR and RES could be a promising preventive and/or therapeutic strategy for the treatment of colon cancer [120]. The effects of polyphenol combinations on cancer cells are summarized in Table 2.

**Table 2:** *In vitro* and *in vivo* antitumoral effects of combinations of polyphenols.

Treatment	*In Vitro* Model	*In Vivo* Model	Antitumoral Effects	Reference
Pterostilbene + quercetin (s)	B16M-F10 melanoma cells (40 µM pterostilbene + 20 µM quercetin)	C57BL/6J mice bearing B16M-F10 cells (20 mg/kg/day of each polyphenol i.v.)	↓ Tumor growth ↓ Metastatic activity ↓ Bcl-2 expression ↑ Mice survival	[105]
Thearubigin + genistein (s)	PC-3 prostate cancer cells (0.125–0.5 µg/mL thearubricin + 5–20 µg/mL genistein)		↓ Cell proliferation ↑ Proportion of cells in G2/M-phase	[106]
Genistein + RES		SV40 rats bearing prostate cancer (83–250 mg/kg/day of each polyphenols p.o)	↓ Tumor growth ↓ IGF-1 expression	[107]
Quercetin + EGCG (a)	PC-3, LNCaP prostate cancer cells (10–20 µM of each polyphenol)	SCID mice bearing LAPC-4 prostate cancer cells (0.2%–0.4% of each polyphenol/day p.o)	↓ Tumor growth ↓ AR expression ↓ PI3K/Akt pathway ↑ Bax/Bcl-2 ratio	[108,109]
CUR + EGCG (s)	A549, NCI-460NSCLC cells (10–20 µM of each polyphenol)	Lung cancer xenograft node mouse model (20 mg/kg/day of each polyphenol i.p.)	↓ Tumor growth ↓ Cyclin D1 and B1 levels	[110]
	MDA-MB-231 breast cancer cells (2–3 µM CUR + 20–25 µM EGCG)	Athymic nude mice implanted with MDA-MB-231 cells (200 mg/kg/day CUR p.o. + 25 mg/kg/day EGCG i.p.)	↓ Tumor volume ↑ Proportion of cells in G2/M-phase	[111]
Arc + CUR + EGCG (s)	LNCaP prostate cancer cells, MCF-7 breast cancer cells (1 μM Arc + 5–10 μM CUR + 40 μM EGCG)		↓ Cell proliferation ↑ Proportion of cells inG0/G1-phase ↑ Bax/Bcl-2 ratio ↓ NF-κB, PI3K/Akt, STAT3 expression	[112]
Luteolin + EGCG (s)	HNSCC and lung cancer cells (10 μM luteolin + 30 μM EGCG)	Athymic nude mice implanted with HNSCC and lung cancer cells (125 mg/kg luteolin + 10 mg/kg EGCG p.o. 5 days a week)	↓ Tumor growth ↑ PARP, caspase-3 cleavage ↑ p53 phosphorylation ↓ Ki-67 expression	[113]
Ellagic acid + quercetin; Ellagic acid + RES; quercetin + RES (s)	MOLT-4 leukemia cells (ellagic acid + quercetin 0–40 μM; Ellagic acid + RES, quercetin + RES 0–140 mM)		↓ Cell proliferation ↑ Caspase-3 activity	[114,115]
RES + CUR	NUB-7, LAN-5, IMR-32, SK-N-BE neuroblastoma cells (0–100 μM CUR + 0–200 μM RES)		↓ Cell proliferation ↑ p53, Bax, p21 expression	[116]
	SJ-RH4, RD/18 rhabdomyosarcoma cells, Saos-2 osteosarcoma cells (6–50 μM of each polyphenol)		↓ Cell proliferation ↑ Bax/Bcl-2 ratio ↓ ERK phosphorylation	[117]
	CAL-27, SCC-15, FaDu, SALTO HNSCCcells (6–50 μM of each polyphenol) (a)	BALB/c mice implanted with SALTO cells (2 mg of each polyphenol in 50 μL of corn oil p.o. thrice weekly)	↓ Tumor growth ↑ PARP cleavage ↑ Bax/Bcl-2 ratio ↓ ERK1/2 phosphorylation ↑ LC3 II expression	[118]
	HCT-116 colon cancer cells (0–50 μM of each polyphenol) (s)	SCID mice implanted with HCT-116 cells (150 mg/kg/day RES + 500 mg/kg/day CUR p.o. for 3 weeks)	↓ Tumor growth ↓ NF-κB, EGFR, IGF-1R activity	[120]

Abbreviations: (s), synergic effect; (a), additive effect; p.o., per os; i.p., intraperitoneally; i.t., intratumorally; i.v., intravenously; s.c., subcutaneously.

## 6. Combinations of Polyphenols and Anticancer Drugs: *In Vitro* and *in Vivo* Antitumoral Effects

Conventional therapies such as chemotherapy and radiotherapy are the gold standard in cancer treatment. Unfortunately, tumor cells can become resistant to these therapies, allowing them to efflux chemotherapeutic agents, modify drug targets by altering expression of genes and proteins involved in carcinogenesis, and increase production of anti-apoptotic proteins such as Bcl-2 and Bcl-Xl [121]. Multiple factors argue for the use of natural polyphenols to enhance the anticancer effects of conventional therapies, including: their ability to modulate different signal transduction pathways involved in carcinogenesis; increasing evidence that combinations of polyphenols significantly counteract tumor growth; and the development of novel polyphenol derivatives with improved bioavailability. Combining polyphenols with conventional therapies may help to overcome drug resistance and reduce the side effects of standard anticancer treatments.

In particular, CUR has demonstrated a potent ability to increase the efficacy of conventional cancer therapies and to chemosensitize cells of colorectal, HNSCC, pancreatic, bladder, and breast tumors [122,123,124,125,126,127,128,129,130]. Shakibadei *et al.* demonstrated that CUR increased the effect of 5-fluorouracil (5-FU) against the colorectal cancer cell lines HCT116 and HCT116+ch3 (complemented with chromosome 3). Notably, pretreatment with CUR reduced IC_50_ values for 5-FU in both cells lines. CUR achieved this result (a) by sensitizing colon cancer cells to treatment with 5-FU, especially by augmenting the induction of apoptosis by 5-FU (favoring mitochondrial degeneration and cytochrome c release, and modulating the expression/cleavage of the pro-apoptotic proteins caspase-8, -9, -3, Bax, and PARP); (b) by down-regulating expression of survival proteins such as cyclin D1; and (c) by modulating NF-κB and PI-3K/Src signaling in both cell lines. It is important to note that the best effect was achieved in HCT116+ch3 cells, suggesting that introduction of chromosome 3 played a crucial role in enhancing sensitivity of HCT116 cell line to treatment with 5-FU and/or CUR. The combination of CUR with conventional chemotherapeutic agents could be a promising strategy for increasing the efficacy of treatments for chemoresistant colon cancer cells [122].

Abuzeid *et al.* studied the effect of a novel CUR analog on cisplatin-sensitive (UM-SCC-74B) and cisplatin-resistant (UM-SCC-29) HNSCC cell lines *in vitro*. FLLL32, a novel small inhibitor derived from CUR, down-regulated the phosphorylated form of STAT3 protein and increased the number of apoptotic cells in both cell lines when used either alone or in combination with cisplatin. In particular, FLLL32 sensitized UM-SCC-29 cells to cisplatin treatment, allowing for a 4-fold reduction in the dose of cisplatin compared to the dose required for cisplatin as monotherapy [123]. CUR also potentiated the anticancer activity of gemcitabine in pancreatic cancer *in vitro* and *in vivo* in an additive manner. *In vitro*, CUR suppressed the growth of tumor cells, enhanced gemcitabine-induced apoptosis, and inhibited the constitutive NF-κB activation of several pancreatic cancer cell lines [124,125].

Lev-Ari *et al.* produced similar results *in vitro* by combining CUR and celecoxib. In this case, CUR synergistically potentiated the pro-apoptotic and antiproliferative effects of celecoxib in pancreatic adenocarcinoma cells [126]. *In vivo*, CUR enhanced the antiproliferative and antiangiogenic effects of gemcitabine in mice bearing orthotopic pancreatic tumors. Mice treated with CUR plus gemcitabine had a significant decrease in tumor volume and significant down-regulation of NF-κB-regulated genes (cyclin D1, c-myc, Bcl-2, Bcl-xL, cellular inhibitor of apoptosis protein-1, COX-2, matrix metalloproteinase, and VEGF) compared to control- and gemcitabine-treated mice [125]. In another study, combination treatment with CUR, raspberry extract (RSE), and neem leaf extract (NLE) effectively induced death/radiosensitization in several human pancreatic cancer cell lines. This three-compound treatment significantly reduced cell viability and increased apoptosis by: (a) enhancing the activity of caspase-3 and -7; (b) modulating the transcription of genes involved in the NF-κB pathway; and (c) inhibiting the NF-κB-DNA-binding activity induced by radiotherapy [127]. These results suggest the potential for these phytochemicals to enhance the effects of chemotherapy and radiotherapy, especially in the treatment of pancreatic cancer with an apoptosis-resistant phenotype [124,125,126,127].

Kamat *et al.* evaluated the capacity of CUR to enhance the anticancer effects of intravesical Bacillus Calmette-Guerin (BCG) in the treatment of bladder cancer. CUR potentiated the apoptotic and antiproliferative effects of BCG on different human bladder cancer cell lines *in vitro* by inhibiting NF-κB activation and up-regulating TNF-related apoptosis-inducing ligand (TRAIL) receptors; these latter are proteins involved in the induction of apoptosis. CUR also potentiated the anticancer effects of BCG in syngeneic CH3 mice implanted with MBT-2 bladder cancer cells. Mice treated with CUR plus BCG showed a significant reduction of tumor growth compared to mice treated with either CUR or BCG alone. This effect was due to the ability of CUR to: (a) inhibit expression of biomarkers of proliferation (Ki-67) and angiogenesis (CD31); (b) enhance BCG-induced apoptosis; (c) reduce cyclin D1, COX-2, c-myc, Bcl-2, and VEGF expression; and (d) suppress the NF-κB pathway in tumor tissue. These promising results suggest that CUR may improve treatments for bladder cancer [128].

CUR also has proven effects in combination with conventional therapies for breast cancer. Kang *et al.* investigated ability of CUR to modulate the effects of paclitaxel on breast cancer *in vitro* or *in vivo.* In the MDA-MB-231 breast cancer cell line *in vitro*, CUR inhibited paclitaxel-induced NF-κB activation by blocking degradation of IκBα, and potentiated the antiproliferative effect of paclitaxel by enhancing induction of apoptosis. Moreover, in athymic NCr-nu/nu mice implanted with MDA-MB-231 cells, combined treatment with 100 mg/kg of CUR plus 7 mg/kg of paclitaxel (a) significantly suppressed tumor growth; (b) markedly reduced tumor cell proliferation rate; (c) improved inhibition of MMP-9 expression; and (d) enhanced apoptosis compared to treatment with CUR or paclitaxel alone. These findings suggest that CUR plus paclitaxel combination may represent a new strategy against breast cancer [129].

In another breast cancer study, Singh *et al.* showed that CUR and RES enhanced the susceptibility of human MCF-7 and MDA-MB-231 breast cancer cell lines to the anti-neoplastic agent Centchroman (CC). Pre-treating breast cancer cells with low-dose RES or CUR potentiated the anticancer activity of CC in both cell lines, thus increasing the number of cells in the sub-G0/G1 phase, disrupting mitochondrial membrane potential, and favoring ROS generation. RES/CUR treatment also enhanced the pro-apoptotic activity of CC by modulating the ROS-mediated JNK/p38 pathway and the mitochondrial pathway in MCF-7 cells. Specifically, the combined treatment promoted phosphorylation of p53, alteration of the Bax/Bcl-2 ratio, and up-regulation of caspase-9 expression. Conversely, RES/CUR treatment induced only caspase-9 expression in MDA-MB-231 cells, suggesting that RES/CUR treatment could increase apoptosis without involving the ROS-mediated JNK/p38 pathway in these cells. The capacity of these two polyphenols to increase the pro-apoptotic activity of CC offers novel opportunities to design new therapies for hormone-dependent breast cancer [130].

RES has been shown to be a multi-targeting compound capable of enhancing the anticancer activity of conventional therapies for several types of cancers. Harikumar *et al.* reported that RES synergized the effect of gemcitabine on human pancreatic cancer cells *in vitro* and *in vivo* by: (a) inhibiting the NF-κB pathway; (b) inhibiting Bcl-2, Bcl-xL, COX-2, cyclin D1, MMP-9, and VEGF expression; and (c) down-regulating the production of markers for angiogenesis (CD31) and cellular proliferation (Ki-67).

*In vivo*, the combination of RES and gemcitabine significantly suppressed the proliferation of MIA PaCa-2 pancreatic cancer cells [131]. RES also potentiated the efficacy of the mTOR inhibitor rapamycin in several breast cancer cell lines in an additive manner, mainly by modulating the Akt signaling transduction pathway. RES inhibited the phosphorylation and activation of the PI3K/Akt pathway, enhancing the sensitivity of breast cancer cells to rapamycin *in vitro* [132].

RES and its metabolites have also been shown to work synergistically with chemotherapeutic agents to inhibit metastasis of human colon cancer cells. Aires *et al.* observed that, among various metabolites tested, RES-3-O-sulfate (R3S) most effectively inhibited growth in the human colon carcinoma cell line SW480 and its derived metastatic cell line SW620 in a time- and dose-dependent manner. Moreover, treatment with RES metabolites in combination resulted in a stronger synergic, time-, and dose-dependent anticancer effect than treatment with RES or R3S alone. RES metabolites blocked colon cancer cells in the S phase of the cell cycle, modulated cyclin and cyclin-dependent kinase expression, and induced apoptosis in a p53-dependent manner. Finally, RES metabolites demonstrated synergic effects with SN38 (irinotecan’s active metabolites used in the treatment of metastatic colon cancer) and oxaliplatin in SW620 cells [133]. Overall these studies support the use of RES and RES metabolites as adjuvants to enhance the anticancer effects of conventional therapies [131,132,133].

Polyphenols present in extra virgin olive oil (EVOO) have shown strong anticancer activity that improves the efficacy of conventional treatments for several types of cancers. Menendez *et al.* reported that the polyphenols in EVOO can reverse acquired resistance to trastuzumab in HER2-overexpressing breast cancer cell lines. Among the polyphenols isolated in EVOO, oleuropein aglycone showed the most potent ability to inhibit breast cancer cell proliferation. SKBR3 cells, which hamper HER2 gene amplification, were about five times more sensitive to the antiproliferative activity of oleuropein aglycone than MCF-7 cells, which are HER2^−^. In addition, when oleuropein aglycone was used in combination with trastuzumab in SKBR3 cells, it enhanced the inhibitory effects of trastuzumab in a dose-dependent manner. This effect was due to its ability to decrease the proteolytic cleavage of the HER2 extracellular domain and suppress HER2 overexpression. Oleuropein aglycone also synergistically enhanced down-regulation of HER2 expression mediated by trastuzumab and reversed acquired resistance to trastuzumab in SKBR3 cells, suggesting its potential use in the treatment of trastuzumab-resistant breast cancer [134].

Suganuma *et al.* conducted two studies evaluating the ability of green tea polyphenols (GTPs) to synergize with chemotherapeutic agents in the treatment of lung cancer [135,136]. The first study showed that (−) epicatechin enhanced: (a) EGCG incorporation into the human lung cancer cell line PC-9; (b) inhibition of cell growth; (c) induction of apoptosis; and (d) EGCG-mediated suppression of TNF-α release. In addition, the combination oftamoxifen and sulindac, which cause apoptosis in human cancer cells, and EGCG synergistically induced apoptosis in PC-9 cells *in vitro* [135]. The same authors also examined the effects of EGCG plus celecoxib, a COX-2 selective inhibitor, on cancer-cell proliferation. These two compounds acted synergistically to induce apoptosis by up-regulating growth arrest and expression of DNA damage-inducible gene 153 (GADD153), as well as activating the MAPK signaling pathway (phosphorylation of ERK1/2 and p38) in human lung cancer cell lines. These findings suggest that the selective inhibition of GADD153 expression by EGCG may be a novel strategy for improving treatments for lung cancer [136].

Similar effects on tumor growth of prostate cancer cells were achieved *in vitro* and *in vivo*, combining green tea polyphenols (GTPs) and other selective inhibitors of COX-2. EGCG worked synergistically with the COX-2 inhibitor NS398 to: (a) arrest cell growth; (b) induce apoptosis by altering the Bax/Bcl-2 ratio and increasing pro-caspase-6 and -9 expression and PARP cleavage; (c) suppress expression of peroxisome proliferator activated receptor-γ (PPAR-γ); and (d) suppress activation of NF-κB in multiple human prostate cancer cell lines *in vitro*. These results were confirmed by *in vivo* experiments. Androgen-sensitive human prostate carcinoma cells (CWR22Rv1) were implanted in athymic nude mice, which were then treated with GTPs (0.1% dissolved in drinking water) and celecoxib (5 mg/kg), a COX-2 inhibitor, alone and in combination. As expected, the combined treatment resulted in a significant suppression of tumor growth compared to treatment with either agent alone. In addition, mice treated with GTPs plus celecoxib had a significant reduction in PSA and IGF-1 levels and a significant increase in serum levels of insulin-like growth factor binding protein-3 (IGFBP-3) levels compared to mice treated with either agent alone [137].

Stearns and Wang reported the additive effects of EGCG and taxanes (paclitaxel and docetaxel) in arresting the growth of human PC-3ML prostate cancer cells *in vitro*. EGCG plus a taxane also had an additive effect on cell death by increasing expression of the pro-apoptotic genes p53, p73, p21, and caspase-3. Moreover, combined treatment with EGCG (228 mg/kg) and paclitaxel (20 mg/kg), injected intraperitoneally, more effectively reduced tumor growth and increased survival rates in CB17 SCID mice implanted with PC-3ML cells than EGCG or paclitaxel alone. Importantly, the combination treatment also suppressed bone metastasis resulting from intravenous injection of PC-3ML cells, suggesting that EGCG may enhance the efficacy of taxanes in the treatment of advanced prostate cancer [138]. Similar results were achieved with the combination of EGCG and doxorubicin (DOX) [139].

The combination of EGCG and DOX has also proven effective in lysing synergistically liver cancer cells. Chen *et al.* observed that EGCG suppressed autophagic activity and blocked proliferation of hepatoma Hep3B cells *in vitro* and *in vivo* in a dose- and time-dependent manner [140]. Moreover, green tea catechins such as ECG and EGCG improved the anticancer activity of DOX in mice transplanted with human chemoresistant liver cancer cells (BEL-7404/DOX) in a dose-dependent manner. When given in combination with DOX, green tea catechins markedly reduced tumor volume, with the best effect seen at the highest dose of EGCG. Green tea catechins significantly raised intracellular accumulation of DOX in BEL-7404/DOX cells *in vitro* and *in vivo*, inhibiting the activity of the P-glycoprotein efflux pump, suggesting that catechins could be employed as adjuvants to subvert resistance to DOX in liver cancer [141].

Luo *et al.* demonstrated that EGCG synergistically sensitized breast cancer cell to paclitaxel *in vitro* and *in vivo*. A dramatic reduction of proliferation and an increase of taxol-induced apoptosis was observed in different breast cancer cell lines *in vitro*, where EGCG potentiated activation of c-Jun *N*-terminal kinases (JNKs) mediated by paclitaxel. EGCG also sensitized breast cancer cells to taxol *in vivo*, significantly inhibiting the growth of transplanted breast cancer cells (4T1) in BALB/c mice [142]. Remarkably, EGCG even potentiated the anticancer activity of chemotherapeutic agents in ovarian and pancreatic cancer [143,144]. Chan *et al.* reported that EGCG enhanced susceptibility to cisplatin and inhibited the growth of ovarian cancer cells through the delivery of hydrogen peroxide (H_2_O_2_). EGCG enhanced the efficacy of cisplatin up to six-fold in the ovarian cancer cell lines SKOV3 and CAOV3, and in the C200, acisplatin-resistant cell line. EGCG also has the unusual ability to kill ovarian cancer cells by increasing levels of intracellular H_2_O_2_, suggesting that increasing oxidative stress may improve the efficacy of chemotherapy in ovarian cancer [143].

Tang *et al.* reported that EGCG potentiated the therapeutic efficacy of gemcitabine and CP690550 (tasocitinib), a STAT3 inhibitor, by modulating the STAT3 pathway in human pancreatic cancer cells. By inhibiting the STAT3-mediated pathway, EGCG blocked the migration and invasive capacity of the pancreatic cancer cell lines AsPC-1 and PANC-1 *in vitro.* In both cell lines, EGCG induced apoptosis by up-regulating caspase-3 activity and enhancing gemcitabine-induced cleavage of caspase-3 and PARP. The synergism of EGCG and CP690550 in inducing apoptosis suggests that EGCG may be a promising candidate for new clinical trials for the treatment of pancreatic cancer [144].

The flavonoids quercetin and genistein have also shown anticancer activity when combined with conventional therapies. Quercetin has proven very effective in enhancing the anticancer activity of chemotherapeutic agents in various types of cancers. Staedler *et al.* investigated the capacity of quercetin to increase the effects of DOX in human breast cancer cell lines *in vitro*. Quercetin potentiated the toxicity of DOX, mainly by reducing cell viability, DNA and protein synthesis, and the invasive capacity of cancer cells. These effects were more pronounced in the highly metastatic MDA-MB-231 cell line than in the less aggressive MCF-7 cell line. In addition, quercetin decreased toxic effects of DOX on non-cancer cells, suggesting a useful role in the treatment of breast cancer [145]. Quercetin has also been shown to enhance the effects of cisplatin. Kuhar *et al.* reported that NSCLC H-520 cells pre-treated with quercetin were more susceptible to cell killing by cisplatin than cells treated with cisplatin alone. Cells treated with quercetin plus cisplatin had an increased rate of apoptosis compared to cells treated with cisplatin alone. This effect was mediated by the ability of quercentin to suppress Bcl-xL expression and increase the Bax/Bcl-2 ratio, caspase-3 activity, and cytochrome c release in mitochondria [146]. Similar effects were reported by Sharma *et al.* on human laryngeal HeP2 cells, where quercetin enhanced cisplatin-mediated apoptosis in a synergic manner, modulated MAPK signaling, and induced pro-apoptotic protein expression while increasing oxidative stress and reducing HSP70 activity [147].

Different studies proved that the soy isoflavone genistein is able to potentiate the anticancer activity of cisplatin and gemcitabine. Compared to cisplatin or gemcitabine alone, the combination of genistein plus cisplatin or genistein plus gemcitabine more effectively reduced proliferation and increased apoptosis in human pancreatic carcinoma cell lines *in vitro* and *in vivo.* Genistein also suppressed cisplatin- and gemcitabine-induced activation of NF-κB in pancreatic carcinoma-bearing mice, suggesting the potential use of this isoflavone as an adjuvant to enhance the effects of chemotherapy in pancreatic cancer [148,149,150]. In a prostate cancer study, Raffoul *et al.* found that pre-treatment with the soy isoflavones genistein, daidzein, and glycitein enhanced cell killing by radiation of PC-3 cells *in vitro.* The soy isoflavones achieved this result by activating apoptosis through up-regulation of Bax expression and PARP cleavage and down-regulation of Bcl-xL and survivin expression*. In vivo*, treatment with soy isoflavones potentiated the inhibition of tumor growth by radiation and stabilized metastasis to para-aortic lymph nodes in a PC-3 orthotopic metastatic mouse model, suggesting a potential role for these compounds in new treatments for prostate cancer [151]. The anticancer effects of polyphenols in combination with anticancer agents are summarized in Table 3.

**Table 3:** *In vitro* and *in vivo* antitumoral effects of polyphenols in combination with anticancer drugs.

Treatment	*In Vitro* Model	*In Vivo* Model	Antitumoral Effects	Reference
CUR + 5-FU	HCT-116 colon cancer cells (5 µM CUR + 0–5 µM 5-FU)		↓ IC_50_ of 5-FU ↑ Cytocrome c release ↑ PARP, caspase-3,-8,-9 cleavage ↓ Cyclin D1 expression ↓ NF-κB, PI-3K/Src activity	[122]
CUR + cisplatin	UM-SCC-74B, UM-SCC-29 HNSCC cells (0.3–5 µM CUR + 3–50 µM cisplatin)		↓ Cell proliferation ↓ STAT3 phosphorylation	[123]
CUR + gemcitabine (a)	P34, Panc-1 pancreatic cancer cells. (10–15 µM CUR + 0.1–0.5 µM gemcitabine)		↓ Cell proliferation ↓ COX-2 and p-ERK1/2expression	[124]
	BxPC-3, MIA PaCa-2, Panc-1 pancreatic cancer cells (10 µM CUR + 50 nM gemcitabine)	Mice bearing pancreatic tumors (1 g/kg/day CUR p.o. + 25 mg/kg gemcitabine i.p. twice weekly)	↓ Tumor growth ↓ NF-κB activity ↓ Cyclin D1, c-myc, Bcl-2,Bcl-xL, COX-2, MMP, VEGF expression	[125]
CUR + celecoxib (s)	P-34, MIA PaCa, Panc-1 pancreatic cancer cells (15 µM CUR + 25 µM celecoxib)		↓ Cell proliferation ↓ COX-2 expression	[126]
CUR + RSE + NLE + radiotherapy	BxPC-3, MIA PaCa-2, Panc-1 pancreatic cancer cells (100 nM CUR + 1 µg RSE+ 0.01% NLE + 10 Gy radiotherapy)		↓ Cell proliferation ↑ Caspase-3,-7 activity ↓ NF-κB activity	[127]
CUR + BCG	MBT-2, 253J-BV, KU-7, RT4V6 bladder cancer cells (0–25 µM CUR + 10^6^ CFU BCG)	Syngeneic mice implanted with MBT-2 cells (1 g/kg/day CUR p.o. + 10^6^ CFU BCG i.t. once weekly)	↓ Tumor growth ↓ NF-κB activity ↑ TRIAL receptors ↓ Ki-67, CD31, cyclin D1, COX-2, c-myc, Bcl-2, VEGF expression	[128]
CUR + paclitaxel	MDA-MB-231breast cancer cells (0.01–10 µM CUR + 0.2–100 µM paclitaxel)	Athymic nude mice implanted with MDA-MB-231 cells (100 mg/kg/day CUR p.o. + 7 mg/kg paclitaxel i.p. weekly)	↓ Tumor growth ↓ NF-κB activity ↓ MMP-9 expression	[129]
RES + CUR + CC	MCF-7, MDA-MB-231 breast cancer cells (10–100 µM RES + 10–30 µM CUR + 10 µM CC)		↑ Proportion of cells in G0/G1-phase ↑ ROS generation ↑ p53 phosphorylation ↑ Bax/Bcl-2 ratio ↑ Caspase-9 expression	[130]
RES + gemcitabine (s)	ASPC-1, MIA PaCa-2, Panc-1 pancreatic cancer cells (10 µM RES + 100 nM gemcitabine)	Athymic nude mice implanted with MIA PaCa-2 cells (40 mg/kg /day RES p.o. + 25 mg/kg gemcitabine i.p. twice weekly)	↓ Tumor growth ↓ NF-κB activity ↓ Cyclin D1, Bcl-2, Bcl-xL, COX-2, MMP-9, VEGF, Ki-67, CD31 expression	[131]
RES + rapamycin (a)	MCF-7, MDA-MB-231, BT-549 breast cancer cells (10–50 µM RES + 0–10,000 nM rapamycin)		↓ Cell proliferation ↓ PI3K/Akt pathway	[132]
RES metabolites + SN38 or oxaliplatin (s)	SW480, SW620 colon cancercells (0–60 µM RES + 50 nM SN38 or 500 nM oxaliplatin)		↓ Cell proliferation ↑ Proportion of cells in S-phase ↑ p53 phosphorylation	[133]
EVOO + trastuzumab (s)	MCF-7, SKBR3 breast cancer cells (50 µM EVOO + 100 µg/mL trastuzumab)		↓ Cell proliferation ↓ HER-2 expression	[134]
EGCG + tamoxifen or sulindac (s)	PC-9 lung cancer cells (75 µM EGCG + 0–20 µM tamoxifen or 0–200 µM sulindac)		↓ Cell proliferation ↓ TNF-α release	[135]
EGCG+ celecoxib (s)	PC-9, A549, ChaGo K-1 lung cancer cells (100 µM EGCG + 1–50 µM celecoxib)		↓ Cell proliferation ↑ GADD153 expression ↑ ERK1/2, p38 phosphorylation	[136]
EGCG + NS38 or celecoxib (s)	LNCaP, PC-3, CWR22Rv1 prostate cancer cells (10–40 µM EGCG + 10 µMNS38)	Athymic nude mice implanted with CWR22Rv1 cells (0.1% EGCG in drinking water/day + 5 mg/kg/day celecoxib i.p. 5 days per week)	↓ Tumor growth ↑ Bax/Bcl-2 ratio ↑ PARP cleavage ↑ Caspase-3, -9 expression ↓ NF-κB activity ↓ PPAR-γ expression ↓ PSA, IGF-1 serum levels	[137]
EGCG + paclitaxel or docetaxel (a)	PC-3ML prostate cancer cells (30 µM EGCG+ 6.25 nM paclitaxel or 3.12 nM docetaxel)	CB17 SCID mice implanted with PC-3ML cells (228 mg/kg/day EGCG + 20 mg/kg paclitaxel i.p.weekly)	↓ Tumor growth ↑ p53, p73, p21, caspase-3 expression ↑ Mice survival rate ↓ Bone metastasis	[138]
EGCG + DOX	IBC-10a, PCa-20a, PC-3ML prostate cancer cells (0–60 µM EGCG + 2 nM or 1–6 µM DOX)	NOD-SCID mice implanted with PC-3ML cells (200 µM EGCG + 2 µM DOX)	↓ Tumor growth ↑ PARP cleavage ↑ Mice survival rate	[139]
ECG + EGCG + DOX	BEL-7404/DOX liver cancer cells (60 mg/mL ECG or 14 mg/mL EGCG + 0.8–2.0 mg/mL DOX)	BALB/c nu/nu mice implanted with BEL-7404/DOX cells (40–160 mg/kg EGCG + 2 mg/kg DOX i.p.)	↓ Tumor growth ↓ IC_50_ of DOX ↓ P-glycoprotein expression	[141]
EGCG + paclitaxel (s)	4T1, MCF-7, MDA-MB-231 breast cancer cells (20 µM EGCG + 2 µM paclitaxel)	BALB/c mice implanted with 4T1 cells (30 mg/kg/day EGCG i.p. + 10 mg/kg paclitaxel i.p. thrice weekly)	↓ Tumor growth ↑ JNK phosphorylation	[142]
EGCG + cisplatin	SKOV3, CAOV3, C200 ovarian cancer cells (0–20 µM EGCG + 1–350 µg/mL cisplatin)		↓ Cell proliferation ↑ H_2_0_2_ levels	[143]
EGCG+ gemcitabine or tasocitinib (s)	AsPC-1, PANC-1 pancreatic cancer cells (0–60 µM EGCG + 0.5 µM gemcitabine or tasocitinib)		↓ Cell proliferation ↓ STAT3 pathway ↓ Cell migration ↑ PARP and caspase-3 cleavage	[144]
Quercetin + DOX	MCF-7, MDA-231 breast cancer cells (5–10 µM quercetin + 10–100 nM DOX)		↓ Cell proliferation ↓ DNA and protein synthesis ↓ Cell invasivity	[145]
Quercetin + cisplatin	H520 NSCLC cells (40 µM quercetin + 5 µg/mL cisplatin)		↑ Apoptotic rate ↑ Bax/Bcl-2 ratio ↑ Caspase-3 activity ↑ Cytochrome c release ↓ Bcl-xL expression	[146]
	HeP2 laryngeal cancer cells (40 µM quercetin + 2.5 µg/mL cisplatin) (s)		↓ Akt phosphorylation ↑ JNK phosphorylation ↑ *c-fos* expression ↑ Bax/Bcl-2 ratio ↓ Bcl-xL, Ki-67 expression ↑ Cytochrome c release ↑ Caspase-8 ,-9 activity ↑ ROS production ↓ HSP70 activity	[147]
Genistein + cisplatin	BxPC-3 pancreatic cancer cells (25 µM genistein + 0.5 µM cisplatin)	SCID mice implanted with BxPC-3 cells (800 µg/kg/day genistein p.o. + 9 mg/kg cisplatin/day i.p.)	↓ Tumor growth ↓ NF-κB activity	[148]
	Panc-28, COLO-357, L3.6pl pancreatic cancer cells (30 µM genistein + 1–2 µM cisplatin)	SCID mice implanted with COLO-357 cells (1 mg/day genistein p.o. + 9 mg/kg cisplatin i.p.)	↓ Tumor growth ↓ NF-κB activity ↓ Bcl-2 , Bcl-xL, MMP-9 expression ↑ PARP and caspase-3 cleavage ↓ Akt phosphorylation ↑ Cytochrome c release	[149]
Genistein + gemcitabine	COLO-357, L3.6pl pancreatic cancer cells (25 µM genistein + 25 nM gemcitabine)	SCID mice implanted with COLO-357 and L3.6pl cells (1 mg/day genistein p.o. + 80 mg/kg/day gemcitabine i.v.)	↓ Tumor growth ↓ NF-κB activity ↑ PARP and caspase-3 cleavage ↑ Cytochrome c release ↓ Bcl-2 , Bcl-xL expression ↓ Akt phosphorylation	[150]
Isoflavones + radiotherapy	PC-3 prostate cancer cells (0–15 µM isoflavones + 3 Gy radiotherapy)	Nude mice implanted with PC-3 cells (1 mg/day isoflavones p.o. + 5 Gy radiotherapy)	↓ Tumor growth ↑ Bax expression ↑ PARP cleavage ↓ Bcl-xL, survivin expression ↓ Metastasis to para-aorticlymph nodes	[151]
Cur-NPs	CAL-27-cisplatin-resistent HNSCC cells (0–80 µM)		↓ Cell proliferation ↑ Bax expression ↑ Caspase-3 ,-9 synthesis ↓ Bcl-2 , MDR1 expression ↑ ROS production	[152]
GLUT1-PEG-PE micelles co-loaded with CUR and DOX	HCT-116 colon cancer cells (7.5–20 µM CUR + 0.1–0.4 µM DOX)	NU/NU nude mice implanted with HCT-116 cells (4 mg/kg/day CUR + 0.4 mg/kg/day DOX i.v.)	↓ Cell viability ↓ Tumor growth ↑ Mice survival	[153]
MPEG-PCL micelles loaded with CUR and DOX (s)	LL/2, MS1 lung cancer cells (0–3 µg/mL CUR and DOX)	C57 mice implanted with LL/2 cells (5mg/kg CUR + 5 mg/kg DOX i.v. every five days)	↓ Tumor growth ↑ Apoptosis ↓ Angiogenesis	[154]
Liposomal CUR + cisplatin	CAL-27, UM-SCC1 HNSCC cells (100 µM CUR + 10–20 µM cisplatin)	Athymic nude mice implanted with HNSCC cells (50 mg/kg CUR i.v. thrice weekly for three weeks + 0.75 µg/mL cisplatin i.p. after 4 weeks)	↓ Tumor growth ↓ Cyclin D1expression ↓ NF-κB pathway ↑ p53 activity	[155]
PLGA-Nano-CUR particles + cisplatin or radiotherapy	cisplatin-resistant A2780CP ovarian cancer cells (2–20 µM CUR + 2.5–40 µM cisplatin; 2–8 µM CUR + 0–4 Gy radiotherapy)		↓ Cell proliferation ↓ Bcl-xL, Mcl-1 expression ↑ PARP, caspase-3, -7, -9 cleavage ↓ β-Catenin activity	[156]

Abbreviations: (s), synergic effect; (a), additive effect; p.o., per os; i.p., intraperitoneally; i.t., intratumorally; i.v., intravenously; s.c., subcutaneously.

## 7. Combinations of Polyphenols in Clinical Trials

Promising preclinical data on the use of combinations of polyphenols or polyphenols and anticancer drugs have spurred interest in using these natural compounds in the clinical setting. Several ongoing and completed clinical trials have reported the safety and efficacy of polyphenols as anticancer agents [157,158,159,160]. However, few clinical trials have evaluated polyphenols in combination with conventional cancer treatments. In a phase I dose-escalation trial combining CUR and docetaxel in advanced and metastatic breast cancer, Bayet-Robert *et al.* demonstrated that the best-tolerated dose of CUR was 6000 mg/day given orally for seven days every three weeks in combination with a standard dose (100 mg/m^2^) of docetaxel given every three weeks for six cycles. This therapeutic protocol proved more effective than treatment with docetaxel alone in reducing tumor marker levels and tumor burden. In addition, concurrent administration of CUR did not increase the side effects of docetaxel, demonstrating the feasibility, safety, and tolerability of this combined treatment. A phase II randomized clinical trial is ongoing to elucidate the mechanism of action by which CUR enhances the efficacy of docetaxel in the treatment of advanced and metastatic breast cancer [161].

Another clinical trial is evaluating the anticancer effects of EGCG in breast cancer patients receiving radiotherapy. Data from this ongoing trial reveal that EGCG in capsule (400 mg three times a day) reduced serum VEGF and HGF levels and suppressed MMP-9/MMP-2 activation, factors associated with the progression and metastasis of breast cancer. When added to a culture medium containing highly aggressive human MDA-MD-231 breast cancer cells, sera from patients treated with EGCG plus radiotherapy strongly suppressed cell viability, arrested the cell cycle at the G0/G1 phase, and induced apoptosis [162].

## 8. Nanotechnology and Polyphenols

There have been few clinical trials of polyphenols in combination with conventional cancer therapies, possibly because the metabolism, stability, drug interactions, side effects, and mechanisms of action of these plant derivatives have not been fully elucidated in humans. In fact, it has been shown that incorrect dosage or route of administration of these phytochemicals may interfere with the activity of conventional therapies and result in harmful effects in humans [14,163].

Nanotechnology may offer a promising solution to these problems. Encapsulating polyphenols in nanoparticles could enhance their biodistribution, solubility, and stability in the human body, while reducing their intense metabolism. Moreover, conjugating nanoparticles containing a specific polyphenol with an appropriate anticancer drug may improve internalization of these natural compounds into cancer cells, leading to improved anticancer activity [164]. Several studies have reported that nanotechnology enhanced ability of CUR to counteract the growth of various tumors. Chang *et al.* found that CUR-loaded nanoparticles (Cur-NPs) selectively reduced the viability of cisplatin-resistant CAL-27 human oral cancer cells (CAR cells) in a dose- and time-dependent manner. Cur-NPs induced intrinsic apoptotic processes (up-regulating Bax, capsase-3, and caspase-9 synthesis and down-regulating expression of multiple drug resistance protein 1 (MDR1) and Bcl-2) and enhanced ROS production in CAR cells [152].

Abouzeid *et al.* used a different combination to counteract the proliferation of HCT-116 cells. They investigated the therapeutic efficacy *in vitro* and *in vivo* of polymeric micelles targeted with an anti-GLUT1 (Glucose Transporter Type 1) antibody (GLUT1-PEG-PE micelles) and co-loaded with CUR and DOX. These micelles showed improved toxicity against HCT116 cells *in vitro*, even at low doses of DOX, compared to non-targeted micelles. Moreover, nude mice injected with HCT116 tumor cells, then treated with GLUT-1-PEG-PE micelles co-loaded with 4 mg/kg of CUR plus 0.4 mg/kg of DOX, displayed greater inhibition of tumor growth and improved survival compared to untreated mice and mice treated with targeted micelles co-loaded with CUR or DOX alone. These promising results suggest a role for these formulations in decreasing glucose uptake, suppressing GLUT1 protein activity, or enhancing delivery of CUR and DOX into colon cancer cells *in vivo* [153].

Similarly, methoxypoly(ethylene glycol)-poly(caprolactone) (MPEG-PCL) micelles loaded with CUR and DOX (Cur-Dox/MPEG-PCL) showed promising anticancer effects and few side effects in the treatment of lung cancer *in vitro* and *in vivo*. These micelles released CUR and DOX slowly into LL/2 and MS1 lung cancer cell lines. In addition, CUR potentiated the anticancer activity of DOX in a synergic manner, indicating a probable synergistic interaction between the two compounds. The ability of CUR-DOX/MPEG-PCL to suppress proliferation of LL/2 cells *in vivo* was investigated using C57 mice bearing LL/2 lung carcinomas. Mice intravenously injected with CUR-DOX/MPEG-PCL micelles containing 5 mg/kg of CUR and 5mg/kg of DOX showed improved inhibition of tumor growth compared to mice treated with micelles containing CUR or DOX alone, indicating *in vivo* synergy between the two compounds. In summary, CUR plus DOX inhibited tumor growth by enhancing apoptosis and suppressing angiogenesis in lung cancer cells, suggesting the potential use of CUR-DOX/MPEG-PCL micelles to improve the treatment of lung cancer in humans [154].

Treatment with liposomal CUR plus cisplatin resulted in greater suppression of tumor growth compared to treatment with cisplatin alone in a murine model of HNSCC. This effect was due to the capacity of CUR and cisplatin to decrease cyclin D1 expression and modulate the NF-κB pathway through reduction of IκBα, phospho-IκBα, and IKKβ expression in HNSCC cells. Cisplatin caused cellular senescence by promoting the activation of p53 protein. These promising *in vivo* results suggest that, in clinical practice, CUR plus cisplatin could potentially reduce the side effects of cisplatin as well as the dose required to inhibit the growth of HNSCC [155].

Yallapu *et al.* investigated the effect CUR nanoparticles on cisplatin-resistant A2780CP ovarian cancer cells. To improve the pharmacokinetics of CUR *in vivo*, a nanoparticle formulation of CUR, conjugated with a monoclonal antibody specific for tumor cells (PLGA-Nano-Cur), was synthesized. PLGA-Nano-Cur particles showed potent antiproliferative activity in A2780CP ovarian cancer cells, supporting the hypothesis that these nanoparticles may enhance delivery of CUR to the tumor site and specifically sensitize chemo- and/or radioresistant cancer cells [156].

The anticancer effects of these polyphenols in combination with anticancer agents are summarized in Table 3.

## 9. Perspective and Conclusions

Polyphenols, compounds ubiquitously expressed in plants, have beneficial effects on human health, including anti-inflammatory, antimicrobial, antiviral, anticancer, and immunomodulatory activities [3,4,5].

Carcinogenesis is a multistep process triggered by genetic alterations that activate multiple signal transduction pathways and cause the progressive transformation of a normal cell into a cancer cell [9]. Signal transduction pathways involved in carcinogenesis often interact with each other, enhancing the oncogenic signals needed to acquire a malignant phenotype [3,10]. Cross-talk between the signaling pathways mediated by ErbB receptors, NF-κB, and the HH/GLI cascade may be the key factor in neoplastic transformation [3].

Due to their ability to modulate the activity of multiple targets involved in carcinogenesis, polyphenols can inhibit the growth of cancer cells [3,90,91,92,93,95,96,97]. Yet despite promising results from *in vitro* studies, in clinical practice, the use of polyphenols as single anticancer agents is limited. This fact is mainly due to their poor bioavailability in the human body. In fact, polyphenols have a poor absorption and biodistribution, but a high metabolism and excretion in the human body, which might hinder the *in vivo* effects of single compounds and affect the effective dose delivered to cancer cells. Although methods for improving the bioavailability of polyphenols have advanced in the last 20 years [4], new strategies are needed to increase the efficacy of polyphenols as anticancer drugs.

One strategy may be to combine different polyphenols with each other, or to use polyphenols in combination with anticancer drugs. Multiple *in vitro* and *in vivo* studies have shown that combinations of polyphenols more effectively inhibit tumor growth than the compounds employed singly. In addition, numerous *in vitro* and *in vivo* studies have shown that polyphenols potentiate the effects of conventional therapies and may help to reduce the effective dose of chemotherapy drugs, overcome drug resistance, and reduce toxicities.

However, there are still only a few clinical trials regarding the use of polyphenols in combination with conventional therapies for cancer treatment. One probable reason could be the fact that metabolism, stability, interaction with other drugs, side effects and mechanisms of action of these plant derivatives have not been fully elucidated in humans. An incorrect administration of these phytochemicals may interfere with the activity of conventional therapies leading to harmful effects in humans [14,163].

A promising solution to overcome these problems could be represented by nanotechnology. In this regard, several preclinical studies reported that the encapsulation of polyphenols in small nanoparticles enhanced their bioavailability and antitumor activity [152,153,154,155,156,164].

The development of nanotechnology to increase bioavailability and antitumoral activities of polyphenols and their synergistic and/or additive effects with conventional anticancer therapies may provide the starting point to improve the rationale for designing new clinical trials to be employed in cancer treatment.
